# Transcutaneous Imiquimod Combined With Anti‐Programmed Cell Death‐1 Monoclonal Antibody Extends the Survival of Mice Bearing Renal Cell Carcinoma

**DOI:** 10.1002/cam4.70966

**Published:** 2025-05-15

**Authors:** Takashi Karashima, Toshihiro Komatsu, Shinkuro Yamamoto, Kaya Atagi, Hatsune Hashida, Hideo Fukuhara, Kenji Tamura, Shingo Ashida, Taro Shuin, Keiko Udaka, Takahiro Shimizu, Motoaki Saito, Nobutaka Shimizu, Keiji Inoue

**Affiliations:** ^1^ Department of Urology Kochi Medical School Nankoku Japan; ^2^ Department of Immunology Kochi Medical School Nankoku Japan; ^3^ Department Pharmacology Kochi Medical School Nankoku Japan; ^4^ Center for Pelvic Floor Kochi Medical School Hospital Nankoku Japan; ^5^ Center for Photodynamic Medicine Kochi Medical School Hospital Nankoku Japan

**Keywords:** IgG, imiquimod, immune checkpoint inhibitor, programmed cell death‐1, renal cell carcinoma, spleen, T cell

## Abstract

**Purpose:**

Imiquimod (IQM), an imidazoquinoline derivative, is an immunomodulator that activates an adaptive immune response. IQM is applied topically for genital warts and actinic keratosis. Programmed cell death‐1 (PD‐1) suppresses activated T cells by binding to programmed cell death‐ligand 1 and programmed cell death‐ligand 2, braking antitumor immunity. Anti‐PD‐1 therapy has been used for various malignant neoplasms including renal cell carcinoma (RCC). Whether combination therapy with transcutaneous administration of IQM cream and intraperitoneal administration of anti‐PD‐1 monoclonal antibody (mAb) suppresses mouse RCC cells growing in subcutaneous tissue was investigated.

**Methods:**

Female BALB/c mice were implanted subcutaneously with 2 × 10^5^ RENCA mouse RCC cells and treated with a transcutaneously applied cream containing IQM and intraperitoneal administration of anti‐PD‐1 mAb beginning 5 days after cell implantation. Tumor burden and survival of the mice were determined. RENCA tumor‐specific IgG production and a minor CD8^+^ T cell subset derived from the spleen of the mice bearing RENCA tumor were detected by flow cytometry. The tumor and spleen weights of mice treated with IQM, anti‐PD‐1 mAb, and their combination were compared.

**Results:**

Combination therapy with IQM and anti‐PD‐1 mAb significantly suppressed tumor growth compared to each monotherapy and prolonged the survival of the mice. The combination therapy produced more RENCA tumor‐specific IgG than either IQM or anti‐PD‐1 mAb alone. The percentage of the CD44^high^CD62L^low^ CD8^+^ T cell subset (effector memory T cells) among splenocytes from mice treated with IQM therapy increased. The CD44^low^CD62L^low^ CD8^+^ T cell subset (pre‐effector‐like T cells) of mice treated with anti‐PD‐1 mAb increased. A negative correlation between tumor and spleen weights was suggested in mice treated with therapies containing IQM.

**Conclusions:**

The present results show that combination therapy with IQM and anti‐PD‐1 mAb might be a promising novel therapeutic strategy for advanced RCC.

**Abbreviations**
CTL: cytotoxic T lymphocyte
Foxp3: forkhead box protein P3
ICI: immune checkpoint inhibitor
IFN‐γ: interferon gamma
IQM: imiquimod
irAEs: immune‐related adverse events
mAb: monoclonal antibody
PD‐1: programmed cell death‐1
RCC: renal cell carcinoma
TKI: tyrosine kinase inhibitor
TLR: toll‐like receptor

## 1 Introduction

Renal cell carcinoma (RCC) is a common neoplasm derived from renal parenchyma. Although RCC is frequently detected incidentally, 24% of patients have distant metastases at the time of diagnosis [1]. Recently, immune checkpoint inhibitors (ICIs) including anti‐programmed death‐1 (PD‐1) antibody have become widely used for RCC patients with distant metastases. PD‐1 protein is expressed on many immune cells, including activated T cells, B cells, natural killer cells, macrophages, and antigen‐presenting cells; it suppresses anticancer immunity by engaging its ligand, programmed cell death‐ligand 1 and programmed cell death‐ligand 2, which is expressed on tumor cells [2]. In patients with RCC, anti‐PD‐1 monoclonal antibody (mAb) has been identified as the first ICI. In a randomized, Phase 3 study of therapy‐experienced patients with advanced RCC, overall survival of the patients treated with the anti‐PD‐1 mAb nivolumab was longer than that of patients treated with the inhibitor of mammalian target of rapamycin everolimus [3].

Imiquimod (IQM) [3‐(2‐methylpropyl)‐3,5,8‐triazatricyclo (7.4.0.02,6) trideca‐1, 2, 4,7,10,12‐hexaen‐7‐amine] is one of the imidazoquinoline derivative agents that was originally used to treat genital warts as a topical cream [4]. IQM is a ligand of toll‐like receptor (TLR)‐7, which induces production of cytokines, including interferon alpha and interleukin‐12, in a variety of immune cell types, leading to the suppression of proliferation of viruses [5]. The antitumor effects of topical IQM therapy have been reported in patients with skin neoplasia, including actinic keratosis [6], basal cell carcinoma [7], and melanoma in situ [8]. Recently, we demonstrated that combination therapy with transcutaneous IQM and oral sorafenib targeting vascular endothelial growth factor receptor, platelet‐derived growth factor receptor, Fms‐like tyrosine kinase 3, and c‐kit suppressed mouse RCC growing in an orthotopic model [9].

The aim of the present study was to determine whether combined transcutaneous administration of IQM and intraperitoneal administration of anti‐PD‐1 monoclonal antibody was effective as a novel therapeutic strategy for mouse RCC. The results of the present study may help to develop a combination therapy that activates natural and adaptive immunity for patients with advanced RCC.

## 2 Materials and Methods

### 2.1 Cell Lines and Culture Conditions

The mouse RCC cell line RENCA was obtained from the American Type Culture Collection (Manassas, VA, USA). RENCA was established from a tumor that arose spontaneously as a renal cortical adenocarcinoma in BALB/c mice. The cells were maintained as monolayer cultures in RPMI‐1640 medium (Wako Pure Chemical Industries Ltd., Osaka, Japan) supplemented with 10% fetal bovine serum (FBS; Thermo Fisher Scientific K.K., Yokohama, Japan). Adherent monolayer cultures were maintained on plastic and incubated at 37°C in a mixture of 5% CO_2_ and 95% air. The cultures were maintained for no longer than 12 weeks following recovery from frozen stocks [9].

### 2.2 Reagents

IQM as Beselna cream, which is often used in cream form as an antiviral agent, was obtained from Mochida Pharmaceutical Co. Ltd. (Tokyo, Japan) [10]. Anti‐PD‐1 mAb was obtained from Ono Pharmaceutical Co. Ltd. (Osaka, Japan).

### 2.3 Animals

A total of 41 female, 6‐week‐old BALB/c Cr Slc mice were obtained from Japan SLC Inc. (Shizuoka, Japan). The mice were housed and maintained under specific‐pathogen‐free conditions and were 6 weeks of age at the time of implantation. The care and use of animals in this study were described in a protocol approved by the Kochi Medical School Animal Care and Use Committee (approval number: P‐00019); the protocol conformed to Japanese guidelines on the ethical use of animals [11]. All efforts were made to minimize the number of experimental animals and their suffering [9].

### 2.4 Implantation of Tumor Cells

To produce subcutaneous tumors, RENCA cells were harvested from subconfluent cultures by brief exposure to 0.25% trypsin and 0.02% EDTA. Trypsinization was stopped with medium containing 10% FBS, and the cells were washed once in serum‐free medium and resuspended in RPMI 1640. Mice were anesthetized with a combination of anesthetics, including 0.3 mg/kg of medetomidine (Nippon Zenyaku Kogyo Co. Ltd., Fukushima, Japan), 4.0 mg/kg of midazolam (Sandoz K.K., Tokyo, Japan), and 5.0 mg/kg of butorphanol (Fujifilm Wako Pure Chemical Co., Osaka, Japan), and 2 × 10^5^ RENCA cells were injected subcutaneously into the right side of the back of each mouse. Before implantation of RENCA cells, mice were prepared by shaving the hair on the back using electric hair clippers. The animals tolerated the experimental procedure well [9, 12].

### 2.5 Therapy

A total of 5 days after implantation of tumor cells, mice were randomized into one of four groups (*n* = 10 to 11 each): (i) daily intraperitoneal administration of phosphate‐buffered saline as control vehicle; (ii) daily cutaneous administration of 40 mg/kg IQM as 5% Beselna cream (16 mg/body); (iii) intraperitoneal administration of anti‐PD‐1 mAb, nivolumab, started 4 days after tumor implantation, starting at 20 mg/kg, followed by 10 mg/kg of nivolumab administered for 6 days; or (iv) combination therapy with IQM and anti‐PD‐1 mAb. The mice were treated for 6 weeks, and the tumor volume was subsequently determined. To avoid the direct effects of IQM against tumors, transcutaneous administration of IQM was applied to the left side of the back of the mice in the absence of subcutaneous tumors.

All mice were prepared by shaving the hair on the back using electric hair clippers, followed by an application of depilatory cream to remove fine hair. The depilatory cream was applied for 1 min to the mouse skin, followed by rinsing with warm water. Hair removal was carried out every 3 days [12].

The experimental period was 150 days, and tumor diameters were measured by a caliper once a week. Tumor volume was calculated using the formula (A × B^2^)/2, where A is a major axis, and B is a minor axis.

### 2.6 Reimplantation of Tumor Cells

In one of the ten mice treated with the combination of IQM and anti‐PD‐1 mAb, the tumor fell off and remitted completely in the initial experiment. The mouse was then reinjected subcutaneously with RENCA cells (1.25 × 10^4^ cells) into the back. As a control, the mouse that did not show tumorigenicity in the initial experiment was also injected with RENCA cells subcutaneously. The mice were not administered any therapy and were monitored for 2 months. Similar experiments were conducted twice.

### 2.7 Flow Cytometry

To detect RENCA tumor‐specific IgG production, 50 μL/body of peripheral blood samples were collected from the tail veins of the mice treated with control vehicle, IQM, anti‐PD‐1 mAb, and the combination (*n* = 5–7 mice in each group). Samples were placed at 37°C and 4°C for one hour each, then centrifuged (1000 × *g*) at 4°C for 10 min. The supernatants were collected, and mixtures of serum samples of each group were collected. RENCA cells (1 × 10^5^ cells) were seeded onto 96‐well plates and treated with the collected serum (100 μL) at 4°C for an hour. The cells were washed with Stain Buffer (BD Pharmingen, Tokyo, Japan), centrifuged, stained with FITC‐conjugated F(ab′)2 of goat antimouse IgG (1:400; Thermo Fisher Scientific, Waltham, MA, USA) at 4°C for 30 min, washed with Stain Buffer, and centrifuged.

To detect minor T cell subsets derived from the spleens of the mice bearing RENCA tumor treated with control vehicle, IQM, anti‐PD‐1 mAb, and the combination, the following antibodies were used: APC‐Cyanine7 antimouse CD8a antibody (1:300; 53–6.7, BioLegend, San Diego, CA, USA); APC antimouse/antihuman CD44 antibody (1:300; IM7, BioLegend); PE antimouse CD62L antibody (1:300; MEL‐14, BioLegend); PE antimouse interferon gamma (IFN‐γ) antibody (1:40; XMG1.2, BioLegend); and Alexa Fluor 647 antihuman/antimouse Granzyme B antibody (1:20; GB11, BioLegend). To remove dead cells, the cells were stained with 7‐amino‐actinomycin D (7‐AAD; 1:20; BD Pharmingen). To fix and permeabilize the cells for staining for IFN‐γ and Granzyme B, BD Cytofix/CytopermFixation/Permeabilization Kit (BD Biosciences, San Jose, CA, USA) was used. The cell suspension was immediately transferred to a measurement tube and quantified using BD LSRFortessa X‐20 flow cytometers (BD Biosciences, Franklin Lakes, NJ, USA). The data were analyzed using FlowJo v10.6 software (BD Biosciences).

### 2.8 Correlation of Tumor and Spleen Weights

To evaluate the correlation between tumor and spleen weights, mice with implanted subcutaneous RENCA tumors were treated with control vehicle, IQM, anti‐PD‐1 mAb, and the combination for 2 weeks, and then tumor and spleen weights were measured.

### 2.9 Statistical Analysis

The differences in tumor volumes were analyzed using Student's *t*‐test. The correlation of tumor and spleen weights was analyzed by Pearson's correlation coefficient analysis. Mouse survival curves were prepared using the Kaplan–Meier method. A value of *p* < 0.05 was considered significant.

## 3 Results

### 3.1 Combination Therapy of IQM With Anti‐PD‐1 mAb Suppressed Subcutaneous RENCA Tumor Growth

**FIGURE 1:**
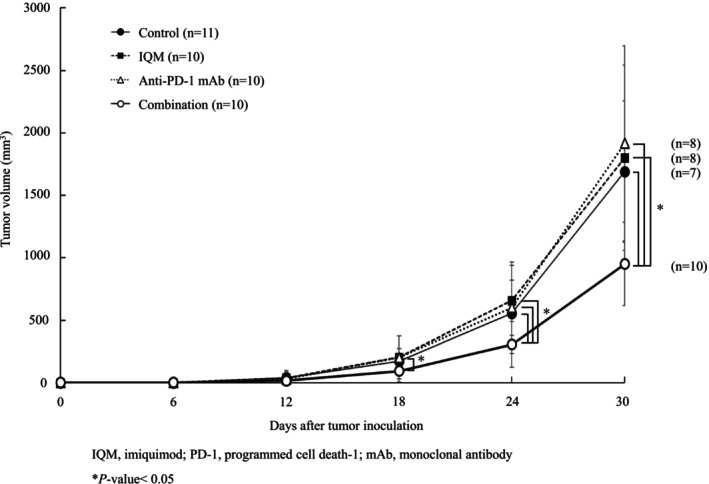
RENCA mouse RCC cells were implanted subcutaneously on the back of BALB/c mice. Therapies were then started 4 days after implantation and continued for 42 days. Tumor volume was measured every 6 days after initiation of therapy with control vehicle, IQM, anti‐PD‐1 mAb, and the combination of IQM and anti‐PD‐1 mAb. The combination therapy significantly suppressed tumor growth when compared with control vehicle, IQM, and anti‐PD‐1 mAb on days 24 and 30 (●, Control; ■, IQM; △, anti‐PD‐1 mAb; ○, combination. *p‐value < 0.05).

To investigate the effects of the therapies (IQM, anti‐PD‐1 mAb, and combination therapy) in the RENCA tumor‐bearing mice, tumor burden and survival curves were evaluated. The combination therapy with IQM and anti‐PD‐1 mAb reduced tumor burden significantly more than control vehicle, IQM, and anti‐PD‐1 mAb at 24 days after therapy was started (0.31 ± 0.18 g vs. 0.56 ± 0.26 g, *p*‐value < 0.02; vs. 0. 66 ± 0.37 g, *p*‐value < 0.01; vs. 0.60 ± 0.28 g, *p*‐value < 0.01, respectively). At 30 days, tumor burden was significantly lower with the combination than with control vehicle and IQM and anti‐PD‐1 mAb alone (0.95 ± 0.33 g vs. 1.69 ± 0.57 g, *p*‐value < 0.01; 1.80 ± 0.78 g, *p*‐value < 0.01; 1.92 ± 0.74 g, *p*‐value < 0.01, respectively, Figure 1).

### 3.2 Combination Therapy of IQM With Anti‐PD‐1 mAb Improved the Survival of Mice Bearing RENCA Tumors; the Mouse Showing Complete Remission With Combination Therapy With IQM and Anti‐PD‐1 mAb Rejected Reimplantation of Tumor Cells

**FIGURE 2:**
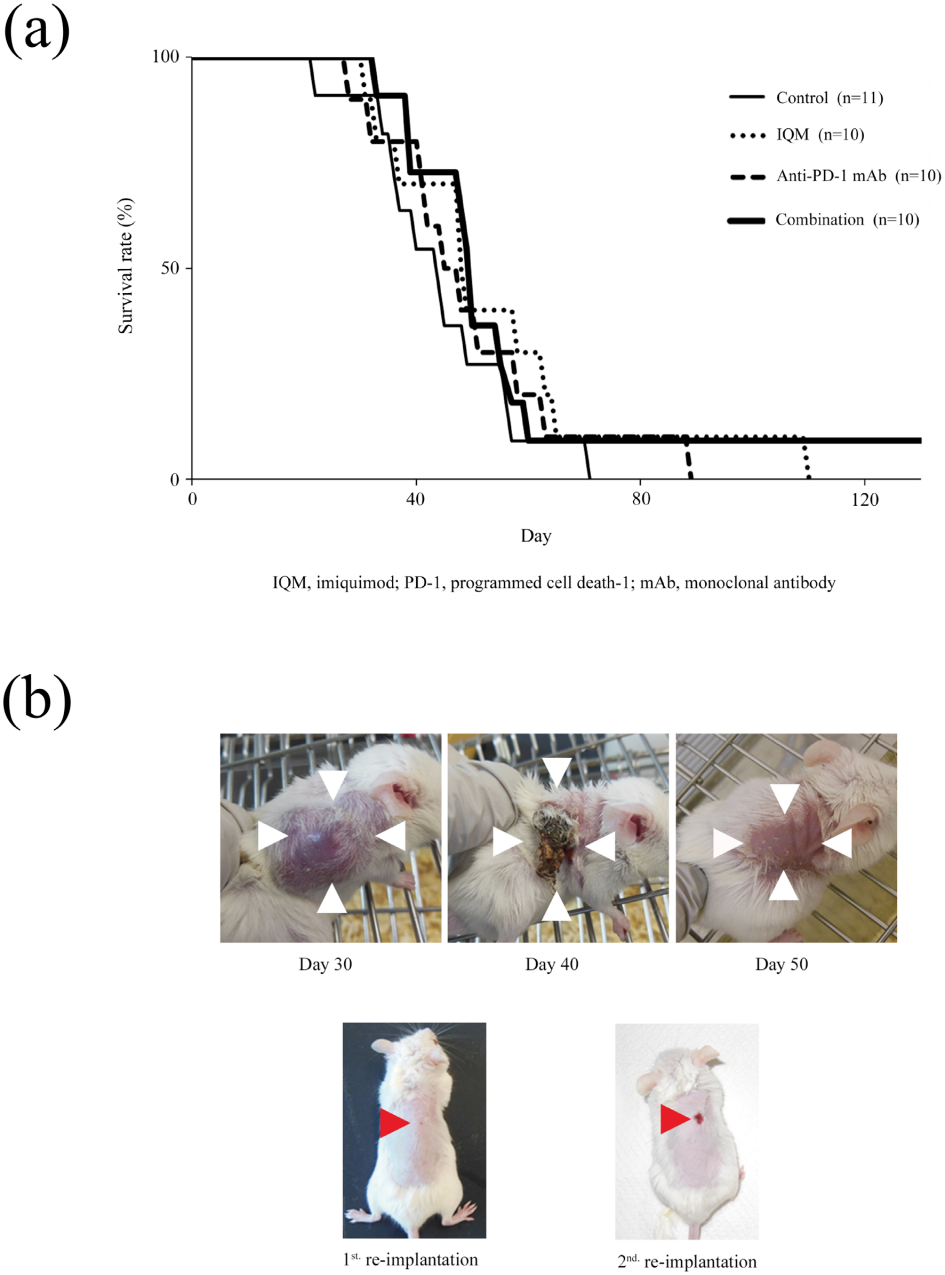
Kaplan–Meier survival curves of the mice treated with control vehicle, IQM, anti‐PD‐1 mAb, and the combination are shown. The mice were continuously observed after the therapy was completed at 42 days. The mice treated with IQM, anti‐PD‐1, and combination therapy showed a tendency for longer survival. Notably, one of the ten mice treated with the combination therapy survived to the end of observation at 150 days after therapy was started (a). Macroscopic findings of the mouse treated with IQM and anti‐PD‐1 mAb showing complete remission (b). The RENCA cells show adequate tumorigenesis at 30 days after combination therapy was started. The tumor is crusting and falling without ulceration at 40 days. At 50 days, the tumor has completely disappeared. RENCA cells were subcutaneously reimplanted in the mouse showing complete remission in the first in vivo animal experiment. The tumor never engrafts during observation without any therapy (1st reimplantation). Furthermore, RENCA cells were reimplanted in the same mouse in the third in vivo experiment. Small crusting without tumor is seen (2nd reimplantation).

Kaplan–Meier survival curves of the mice treated with control vehicle, IQM, anti‐PD‐1 mAb, and the combination were evaluated. The mice treated with IQM, anti‐PD‐1, and combination therapy tended to have longer survival, although the curves did not differ significantly. Notably, one of ten mice treated with combination therapy survived to the end of observation at 150 days after the start of therapy (Figure 2a). Representative macroscopic findings of the mouse treated with combination therapy are presented in Figure 2b. Tumorigenesis was obvious at 30 days, the tumor was spontaneously debrided at 40 days after therapy started, and it disappeared at 50 days.

RENCA cells were reimplanted in the mouse showing complete remission (Figure 2b) and the mouse without tumor engraftment as a control of the initial animal experiment. The mouse showing complete remission rejected implantation of RENCA cells (Figure 2c: 1st reimplantation). Furthermore, RENCA cells were reimplanted in the mouse that rejected tumorigenesis in the 1st reimplantation animal experiment. The mouse rejected the RENCA cells again (Figure 2c: 2nd reimplantation). In the 1st and 2nd reimplantation‐animal experiments, all mice did not have any therapy.

### 3.3 Combination Therapy Induced Tumor‐Specific IgG Production

**FIGURE 3:**
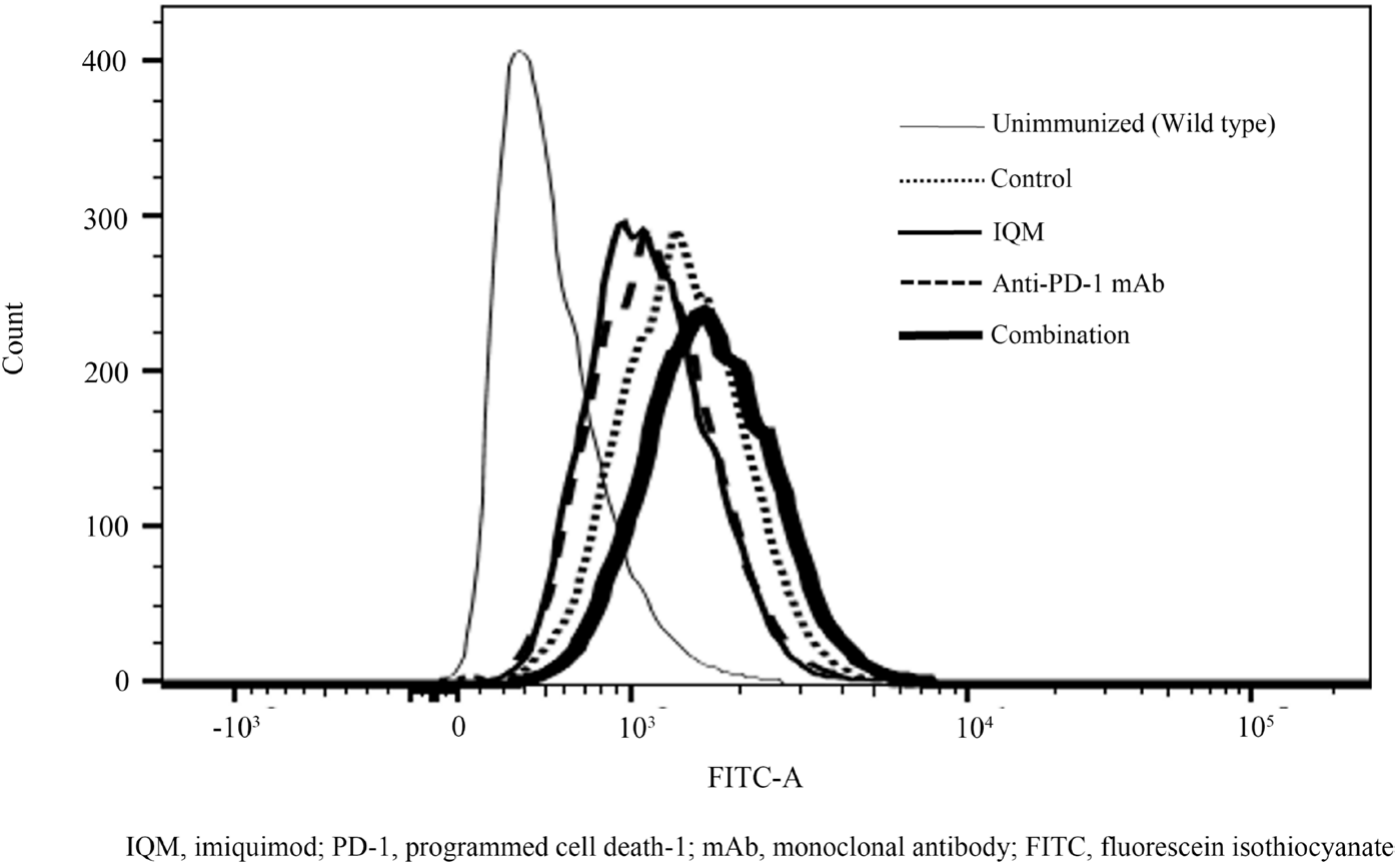
RENCA tumor‐specific IgG production in the serum of mice treated with control vehicle, IQM, anti‐PD‐1 mAb, and the combination was determined by flow cytometry. IgG production is shown in the histogram. The IgG production of the mice without tumor (unimmunized) is shown as the gray curve in the histogram. The curve of combination therapy (black bold curve) is shifted to the far right, meaning that the IgG production of the mice treated with the combination is higher than that of the mice treated with control vehicle (black dotted curve), IQM (black curve), and anti‐PD‐1 mAb (black broken curve). The mice bearing RENCA tumors produced tumor‐specific IgG, even those treated with control vehicle.

The RENCA tumor‐specific IgG production of the mice treated with control vehicle, IQM, anti‐PD‐1 mAb, and the combination in the 1st animal experiment was evaluated by flow cytometry of mouse serum. IgG production was higher in the mice treated with the combination of IQM and anti‐PD‐1 mAb than in the mice treated with control vehicle, IQM, and anti‐PD‐1 mAb (Figure 3).

### 3.4 Combination Therapy Induced the CD44^high^CD62L^low^ CD8 ^+^ T Cell Subset (Effector Memory T Cells) and the CD44^low^CD62L^low^ CD8 ^+^ T Cell Subset (Pre‐effector‐Like T Cells)

**FIGURE 4:**
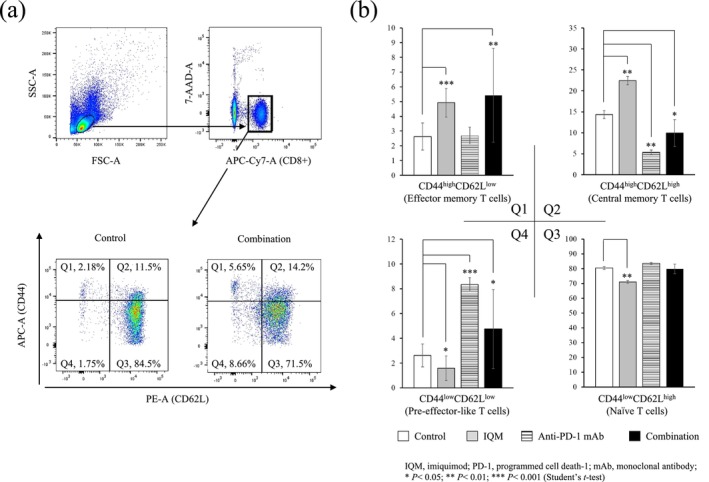
Flow cytometry gating strategy to identify minor CD8^+^ T cell subset of splenocytes from the mice bearing RENCA tumor (a). Representative dot plots showing the expressions of CD44 and CD62L on CD8^+^ T cells from the splenocytes of mice treated with control vehicle and combination (a). Based on the expressions of CD44 and CD62L, four subsets were gated: CD44^high^CD62L^low^ subset (Q1, effector memory T cells); CD44^high^CD62L^high^ subset (Q2, central memory T cells); CD44^low^CD62L^high^ subset (Q3, naïve T cells); and CD44^low^CD62L^low^ subset (Q4, pre‐effector‐like T cells). Numbers in the plots indicate proportions of gated cell populations. Comparison of the percentage of minor CD8^+^ T cell subsets in the therapies with control vehicle, IQM, anti‐PD‐1 mAb, and combination therapy (b). The IQM increases the Q1 and Q2 subsets; the anti‐PD‐1 mAb increases the Q4 subset; the combination of IQM and anti‐PD‐1 mAb increases both the Q1 and Q4 subsets, compared to the control vehicle.

Comparative analysis of CD8^+^ T cell subsets in splenocytes from the mice treated with combination therapy showed that the percentage of the CD44^high^CD62L^low^ CD8^+^ T cell subset (Q1, effector memory T cells) increased with the CD44^low^CD62L^low^ subset (Q4, pre‐effector‐like T cells). The mice treated with IQM showed an increased percentage of the Q1 and CD44^high^CD62L^high^ subsets (Q2, central memory T cells). In addition, anti‐PD‐1 mAb increased the percentage of the Q4 subset (Figure 4).

### 3.5 Combination Therapy Induced the CD8 ^+^ T Cell Subset Expressing IFN‐γ and/or Granzyme B

**FIGURE 5:**
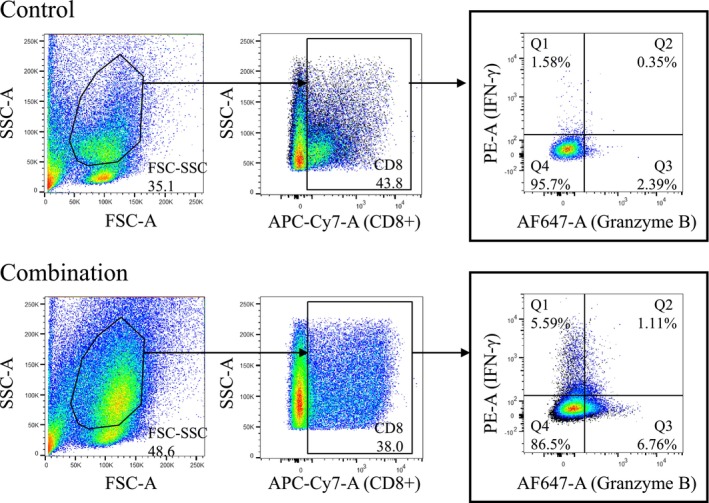
Representative dot plots of flow cytometry showing the expressions of IFN‐γ and Granzyme B on CD8^+^ T cells from the splenocytes of mice treated with control vehicle and combination therapy. Numbers in the plots indicate proportions of gated cell populations. The combination of IQM and anti‐PD‐1 mAb increases the IFN‐γ and/or the Granzyme B subsets (Q1, Q2, and Q3) compared with the control vehicle.

Whether the CD8^+^ T splenocytes expressing IFN‐γ and Granzyme B were induced by the combination therapy with IQM and anti‐PD‐1 mAb was examined by flow cytometry (Figure 5). The proportions of gated cell populations with IFN‐γ and/or Granzyme B‐positive CD8^+^ T cell subsets (Q1, Q2, and Q3) with combination therapy were higher than with the control vehicle (Figure 5).

### 3.6 Negative Correlation of Tumor and Spleen Weights of Mice Treated With Therapies Containing IQM

**FIGURE 6:**
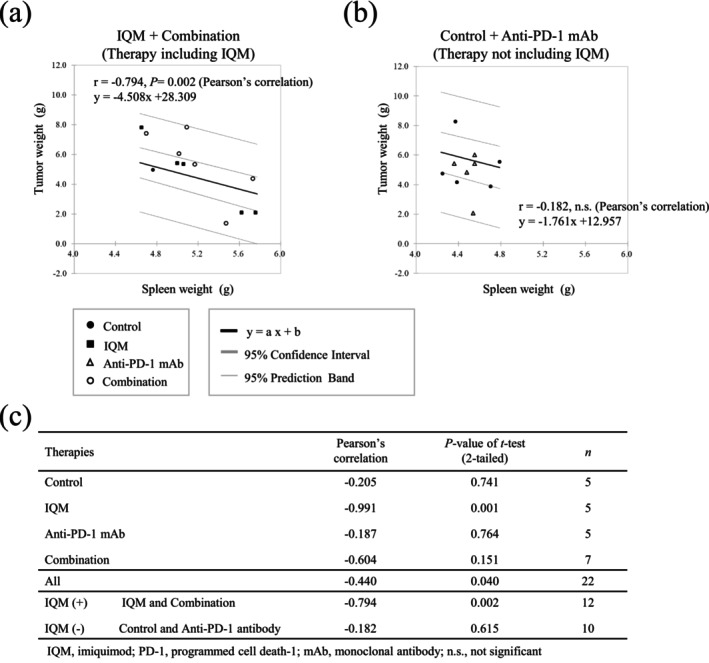
Correlations of tumor and spleen weights in the mice treated with the therapies containing IQM (a) and without IQM (b). The therapies containing IQM (IQM and Combination) show a significantly negative correlation between tumor and spleen weights (Pearson's correlation coefficient = −0.794, *p*‐value = 0.002), whereas the mice treated without IQM (control vehicle and anti‐PD‐1 mAb) do not. Pearson's correlation coefficient and *p*‐values of individual therapies are shown in the lower table (c).

The tumor and spleen weights of mice treated with therapies containing IQM (IQM alone and combination) showed a significantly negative correlation, whereas the mice treated without IQM (control vehicle and anti‐PD‐1 mAb alone) did not. Pearson's correlation coefficient for the therapies containing IQM was −0.794 (*P*‐value = 0.002), whereas it was −0.182 for the therapies not containing IQM. Pearson's correlation coefficient and *P*‐values of individual therapies are shown in the lower part of Figure 6.

## 4 Discussion

The cancer immunity cycle is divided into seven major steps. TLR agonists induce tumor antigen presentation by antigen‐presenting cells/dendritic cells at the second step after releasing tumor antigens as the first step. Anti‐PD‐1 therapies promote cytotoxic T lymphocytes (CTLs) killing tumor cells locally in the tumor in the seventh step in the effector phase [13]. Therefore, the novel therapeutic strategy of the combination with IQM, a TLR‐7 agonist, and nivolumab, an anti‐PD‐1 mAb, efficiently rotates the cancer immune cycle by affecting the second and seventh steps of the cancer immunity cycle, respectively. Our previous studies demonstrated that the major anti‐RENCA tumor activity of IQM therapy involved CTLs [9, 12]. The present data also demonstrated that combination therapy with IQM and the anti‐PD‐1 mAb produced more RENCA‐specific IgG than IQM or anti‐PD‐1 mAb alone (Figure 3). In the in vivo animal experiment, one of the 10 mice treated with combination therapy showed that the tumor was shed and disappeared, resulting in prolonged survival (Figure 2). In addition, the two‐time rapid rejection of reimplantation of RENCA cells supports the development of tumor‐specific IgG immunity, which might induce antibody‐dependent cellular cytotoxicity (ADCC) with natural killer cells [14]. Further investigation of ADCC by the combination therapy of IQM and anti‐PD‐1 mAb might lead to an efficient novel therapeutic strategy.

A recent study reported that the CD44^low^CD62L^low^ CD8^+^ T cell subset (pre‐effector‐like T cells) differentiates from the CD44^low^CD62L^high^ CD8^+^ T cell subset (naïve T cells) and further matures into the CD44^high^CD62L^low^ subset (effector memory T cells) critical to antitumor activity [15]. The present comparative analysis of CD8^+^ T cell subsets in splenocytes from the mice showed that anti‐PD‐1 mAb alone induced the CD44^low^CD62L^low^ CD8^+^ T cell subset (Q4, pre‐effector‐like T cells) differentiated from the CD44^low^CD62L^high^ subset (Q3, naïve T cells), whereas IQM alone induced the CD44^high^CD62L^low^ (Q1, effector memory T cells) and the CD44^high^CD62L^high^ subsets (Q2, central memory T cells). Furthermore, the combination therapy of IQM and anti‐PD‐1 mAb induced both Q1 and Q4 subsets, suggesting that the combination therapy efficiently induced the Q1 from the Q3 via the Q4 subsets (Figure 4). To detect the direct cytotoxic activity of CD8^+^ T cells derived from the spleens of mice bearing RENCA tumor treated with the combination therapy with IQM and anti‐PD‐1 mAb, flow cytometry was performed using anti‐IFN‐γ and Granzyme B antibodies. The combination therapy induced IFN‐γ and/or Granzyme B‐positive CD8^+^ T cells compared with the control vehicle (Figure 5).

Suppressor immunocytes are important for understanding the immune microenvironment. The numbers of CD4^+^ and forkhead box protein P3 (Foxp3)^+^ T cells infiltrating and accumulating in the RENCA tumor were assessed by immunohistochemical staining with anti‐CD4 and Foxp3 antibodies. The number of positive cells was counted in 10 fields of each tumor to calculate the average with standard deviation. The number of CD4^+^ cells in the tumor treated with the combination therapy of IQM and anti‐PD‐1 mAb was significantly higher than with the control vehicle. The number of Foxp3+ cells in the tumor treated with the combination therapy was significantly lower than with the control vehicle. These data suggested that the combination therapy might induce infiltration and accumulation of effector immunocytes and reduce regulatory T cells (Figure S1).

The present data for the relationship between tumor and spleen weights showed a negative correlation in mice treated with therapies containing IQM. In addition, IQM therapy might increase the number of lymphocytes and fuse lymph follicles in the spleen, with resultant splenomegaly, leading to tumor suppression (Figures 6 and S2).

Recently, combination immunotherapy, such as ICIs with tyrosine kinase inhibitors (TKIs), has been approved for patients with advanced RCCs on the basis of the results of large clinical trials such as KEYNOTE‐426 and JAVELIN Renal 101 [16, 17]. We previously demonstrated that combination therapy with transcutaneous IQM and oral TKI suppressed RENCA tumors growing in an orthotopic mouse model [9]. A phase II clinical trial of combination therapy involving IQM and anti‐PD‐1 mAb for advanced cancers began in 2024 in China [18]. Therefore, a triplet combination with IQM, TKIs, and ICIs may be a promising novel strategy. However, we need to pay careful attention to severe immune‐related adverse events (irAEs). In the present study, 42 days after tumor inoculation, four mice treated with combination therapy died rapidly (Figure 2a). The reasons may include severe irAEs or the therapy was stopped at 42 days after it was started. Although macroscopic severe organ damage was not observed in the brain, lung, heart, liver, kidney, or intestine of the mice before cancer death, further examinations for irAEs caused by the combination therapy are urgently needed.

In summary, combination therapy with transcutaneous IQM and i.p. anti‐PD‐1 mAb produced beneficial effects against mouse RCC. Therefore, the combination of IQM and anti‐PD‐1 mAb may represent a promising therapeutic strategy for patients with advanced RCC and warrants further investigation. However, additional preclinical studies are necessary.

## Author Contributions

**Takashi Karashima:** conceptualization (lead), data curation (lead), formal analysis (lead), funding acquisition (lead), investigation (lead), methodology (lead), project administration (lead), supervision (supporting), validation (lead), visualization (lead), writing – original draft (lead), writing – review and editing (supporting). **Toshihiro Komatsu:** data curation (supporting), methodology (supporting), writing – review and editing (supporting). **Shinkuro Yamamoto:** data curation (supporting), formal analysis (supporting). **Kaya Atagi:** data curation (supporting). **Hatsune Hashida:** data curation (supporting), formal analysis (supporting), methodology (supporting), visualization (supporting). **Hideo Fukuhara:** data curation (supporting). **Kenji Tamura:** data curation (supporting). **Shingo Ashida:** data curation (equal). **Taro Shuin:** conceptualization (supporting), supervision (supporting). **Keiko Udaka:** investigation (supporting), supervision (supporting). **Takahiro Shimizu:** supervision (supporting). **Motoaki Saito:** supervision (supporting). **Nobutaka Shimizu:** supervision (supporting). **Keiji Inoue:** supervision (supporting).

## Disclosure

Additional declarations for articles in life science journals that report the results of studies involving humans and/or animals: The care and use of animals in this study were described in a protocol approved by the Kochi Medical School Animal Care and Use Committee; the protocol conformed to Japanese guidelines on the ethical use of animals.

## Ethics Statement

This study was approved by the Kochi Medical School Animal Care and Use Committee. Approval number was P‐00019.

## Consent

The authors have nothing to report.

## Conflicts of Interest

The authors declare no conflicts of interest.

## Supporting information


**Figure S1.** We assessed the number of CD4+ and forkhead box protein P3 (Foxp3)+ T cells infiltrating and accumulating in the RENCA tumor by immunohistochemical staining with anti‐CD4 and Foxp3 antibodies (1:50, D7D2Z and 1:100, D6O8R, respectively. CellSignaling, Massachusetts, USA). The number of positive cells was counted in 10 fields each tumor for calculation of the average with standard deviation. The number of CD4^+^ cells in the tumor treated with the combination therapy of IQM and anti‐PD‐1 mAb was significantly higher than the control vehicle (p* = 0.043). The number of Foxp3^+^ cells in the tumor treated with the combination therapy was significantly lower than the control vehicle (p** = 0.032). Representative microscopic findings with high power field (×40) were shown. Red circles were described positive cells of CD4 and Foxp3 immunohistochemical staining.

**Figure S2.** Macroscopic findings of spleens and subcutaneous tumors, and microscopic findings of spleens in mice treated with control vehicle, IQM, anti‐PD‐1 mAb, and the combination of IQM and anti‐PD‐1 mAb. The spleens in the mice treated with the therapies containing IQM are swollen, whereas the tumor growth is suppressed. Hematoxylin and eosin staining of the spleens in the mice treated with the therapies containing IQM shows proliferation of lymphocytes, fusion of lymphoid follicles, and an increased proportion of white pulp.

## Data Availability

Requests for the study materials and dataset used to support the conclusions of this article should be directed to the corresponding author.
