# Supplementary material for: Transcutaneous Imiquimod Combined With Anti‐Programmed Cell Death‐1 Monoclonal Antibody Extends the Survival of Mice Bearing Renal Cell Carcinoma
Source: Cancer Med. 2025 May 15;14(10):e70966. doi: 10.1002/cam4.70966 (PMC12079644; doi:10.1002/cam4.70966)
Supplement: Supplementary file 1 — Figure S1. We assessed the number of CD4+ and forkhead box protein P3 (Foxp3)+ T cells infiltrating and accumulating in the RENCA tumor by immunohistochemical staining with anti‐CD4 and Foxp3 antibodies (1:50, D7D2Z and 1:100, D6O8R, respectively. CellSignaling, Massachusetts, USA). The number of positive cells was counted in 10 fields each tumor for calculation of the average with standard deviation. The number of CD4+ cells in the tumor treated with the combination therapy of IQM and anti‐PD‐1 mAb was significantly higher than the control vehicle (p* = 0.043). The number of Foxp3+ cells in the tumor treated with the combination therapy was significantly lower than the control vehicle (p** = 0.032). Representative microscopic findings with high power field (×40) were shown. Red circles were described positive cells of CD4 and Foxp3 immunohistochemical staining. [file CAM4-14-e70966-s001.pptx]

## Slide 1
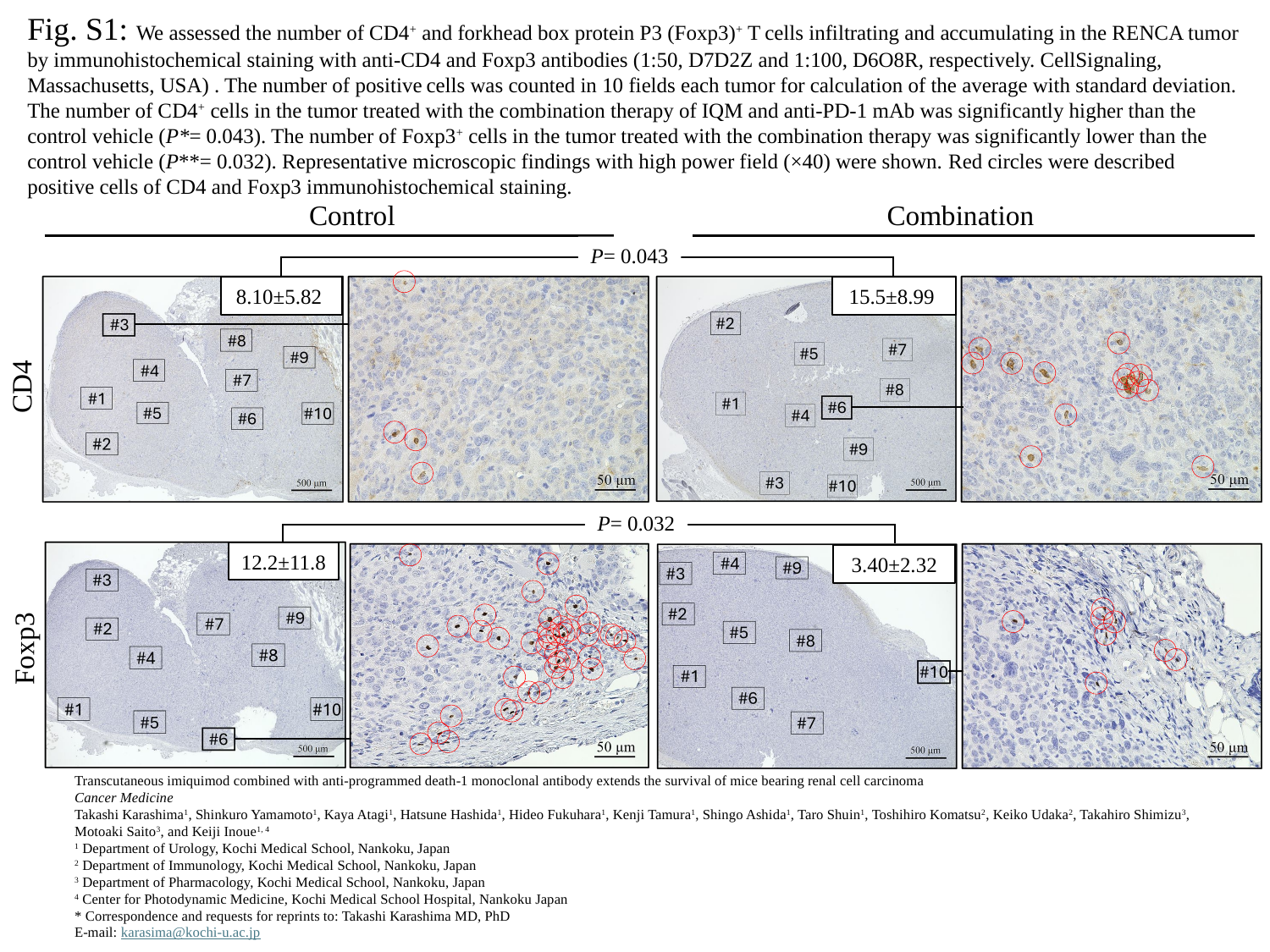

Fig. S1: We assessed the number of CD4+ and forkhead box protein P3 (Foxp3)+ T cells infiltrating and accumulating in the RENCA tumor by immunohistochemical staining with anti-CD4 and Foxp3 antibodies (1:50, D7D2Z and 1:100, D6O8R, respectively. CellSignaling, Massachusetts, USA) . The number of positive cells was counted in 10 fields each tumor for calculation of the average with standard deviation. The number of CD4+ cells in the tumor treated with the combination therapy of IQM and anti-PD-1 mAb was significantly higher than the control vehicle (P*= 0.043). The number of Foxp3+ cells in the tumor treated with the combination therapy was significantly lower than the control vehicle (P**= 0.032). Representative microscopic findings with high power field (×40) were shown. Red circles were described positive cells of CD4 and Foxp3 immunohistochemical staining.
Control
Combination
P= 0.043
15.5±8.99
8.10±5.82
CD4
P= 0.032
12.2±11.8
3.40±2.32
Foxp3
Transcutaneous imiquimod combined with anti-programmed death-1 monoclonal antibody extends the survival of mice bearing renal cell carcinoma
Cancer Medicine
Takashi Karashima1, Shinkuro Yamamoto1, Kaya Atagi1, Hatsune Hashida1, Hideo Fukuhara1, Kenji Tamura1, Shingo Ashida1, Taro Shuin1, Toshihiro Komatsu2, Keiko Udaka2, Takahiro Shimizu3, Motoaki Saito3, and Keiji Inoue1, 4
1 Department of Urology, Kochi Medical School, Nankoku, Japan
2 Department of Immunology, Kochi Medical School, Nankoku, Japan
3 Department of Pharmacology, Kochi Medical School, Nankoku, Japan
4 Center for Photodynamic Medicine, Kochi Medical School Hospital, Nankoku Japan
* Correspondence and requests for reprints to: Takashi Karashima MD, PhD
E-mail: karasima@kochi-u.ac.jp
