# Supplementary material for: Transcutaneous Imiquimod Combined With Anti‐Programmed Cell Death‐1 Monoclonal Antibody Extends the Survival of Mice Bearing Renal Cell Carcinoma
Source: Cancer Med. 2025 May 15;14(10):e70966. doi: 10.1002/cam4.70966 (PMC12079644; doi:10.1002/cam4.70966)
Supplement: Supplementary file 2 — Figure S2. Macroscopic findings of spleens and subcutaneous tumors, and microscopic findings of spleens in mice treated with control vehicle, IQM, anti‐PD‐1 mAb, and the combination of IQM and anti‐PD‐1 mAb. The spleens in the mice treated with the therapies containing IQM are swollen, whereas the tumor growth is suppressed. Hematoxylin and eosin staining of the spleens in the mice treated with the therapies containing IQM shows proliferation of lymphocytes, fusion of lymphoid follicles, and an increased proportion of white pulp. [file CAM4-14-e70966-s002.pptx]

## Slide 1
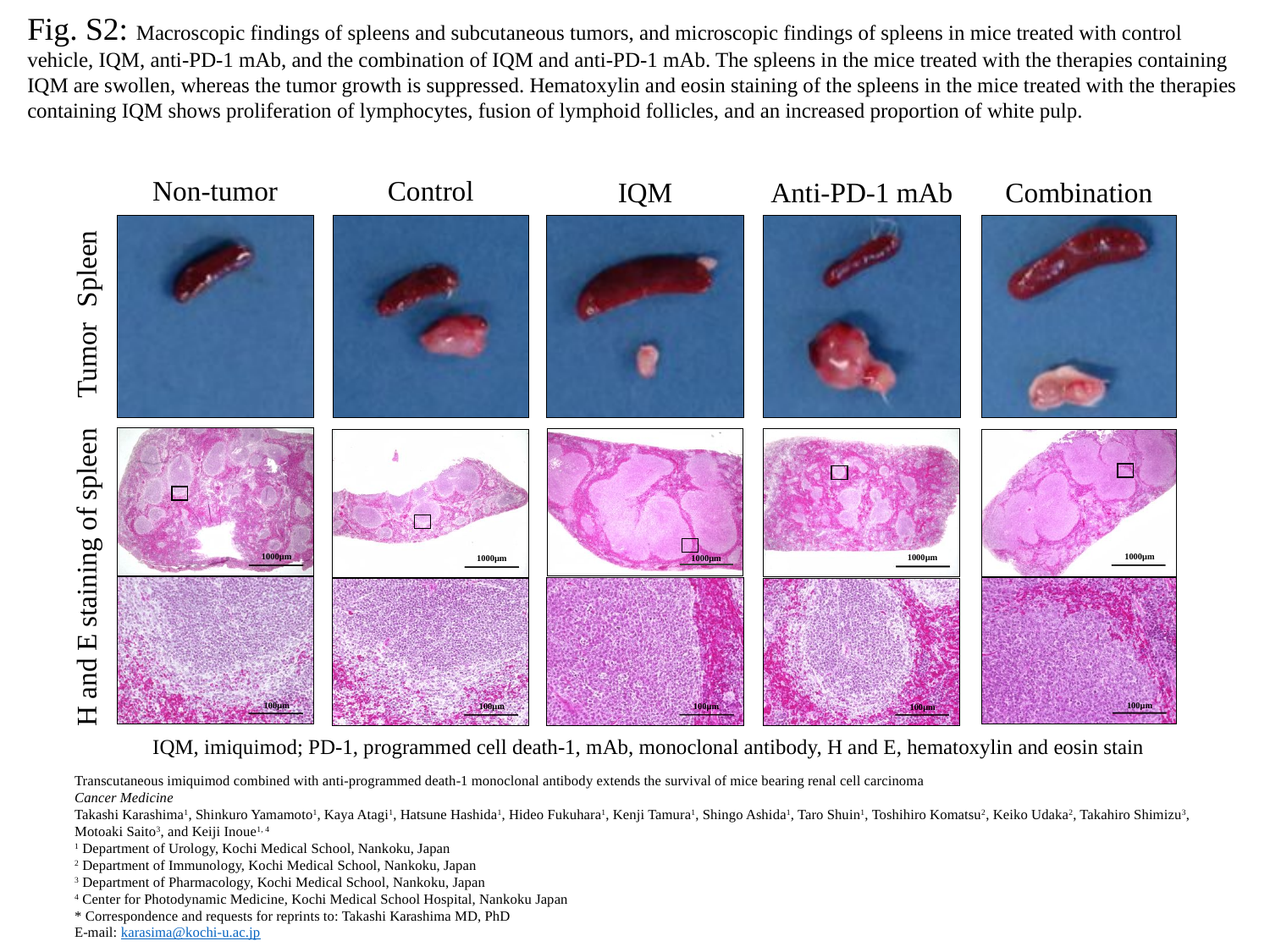

Fig. S2: Macroscopic findings of spleens and subcutaneous tumors, and microscopic findings of spleens in mice treated with control vehicle, IQM, anti-PD-1 mAb, and the combination of IQM and anti-PD-1 mAb. The spleens in the mice treated with the therapies containing IQM are swollen, whereas the tumor growth is suppressed. Hematoxylin and eosin staining of the spleens in the mice treated with the therapies containing IQM shows proliferation of lymphocytes, fusion of lymphoid follicles, and an increased proportion of white pulp.
Control
Non-tumor
Combination
IQM
Anti-PD-1 mAb
Spleen
Tumor
1000μm
1000μm
1000μm
1000μm
1000μm
H and E staining of spleen
100μm
100μm
100μm
100μm
100μm
IQM, imiquimod; PD-1, programmed cell death-1, mAb, monoclonal antibody, H and E, hematoxylin and eosin stain
Transcutaneous imiquimod combined with anti-programmed death-1 monoclonal antibody extends the survival of mice bearing renal cell carcinoma
Cancer Medicine
Takashi Karashima1, Shinkuro Yamamoto1, Kaya Atagi1, Hatsune Hashida1, Hideo Fukuhara1, Kenji Tamura1, Shingo Ashida1, Taro Shuin1, Toshihiro Komatsu2, Keiko Udaka2, Takahiro Shimizu3, Motoaki Saito3, and Keiji Inoue1, 4
1 Department of Urology, Kochi Medical School, Nankoku, Japan
2 Department of Immunology, Kochi Medical School, Nankoku, Japan
3 Department of Pharmacology, Kochi Medical School, Nankoku, Japan
4 Center for Photodynamic Medicine, Kochi Medical School Hospital, Nankoku Japan
* Correspondence and requests for reprints to: Takashi Karashima MD, PhD
E-mail: karasima@kochi-u.ac.jp
